# Reduction of Adipose Tissue Mass by the Angiogenesis Inhibitor ALS-L1023 from *Melissa officinalis*

**DOI:** 10.1371/journal.pone.0141612

**Published:** 2015-11-23

**Authors:** Byung Young Park, Hyunghee Lee, Sangee Woo, Miso Yoon, Jeongjun Kim, Yeonhee Hong, Hee Suk Lee, Eun Kyu Park, Jong Cheon Hahm, Jin Woo Kim, Soon Shik Shin, Min-Young Kim, Michung Yoon

**Affiliations:** 1 Department of Biological Sciences, Korea Advanced Institute of Science and Technology (KAIST), Daejeon, Korea; 2 AngioLab, Inc., Daejeon, Korea; 3 Department of Biomedical Engineering, Mokwon University, Daejeon, Korea; 4 Department of Formula Sciences, College of Oriental Medicine, Dongeui University, Busan, Korea; University of Louisville, UNITED STATES

## Abstract

It has been suggested that angiogenesis modulates adipogenesis and obesity. This study was undertaken to determine whether ALS-L1023 (ALS) prepared by a two-step organic solvent fractionation from *Melissa* leaves, which exhibits antiangiogenic activity, can regulate adipose tissue growth. The effects of ALS on angiogenesis and extracellular matrix remodeling were measured using *in vitro* assays. The effects of ALS on adipose tissue growth were investigated in high fat diet-induced obese mice. ALS inhibited VEGF- and bFGF-induced endothelial cell proliferation and suppressed matrix metalloproteinase (MMP) activity *in vitro*. Compared to obese control mice, administration of ALS to obese mice reduced body weight gain, adipose tissue mass and adipocyte size without affecting appetite. ALS treatment decreased blood vessel density and MMP activity in adipose tissues. ALS reduced the mRNA levels of angiogenic factors (VEGF-A and FGF-2) and MMPs (MMP-2 and MMP-9), whereas ALS increased the mRNA levels of angiogenic inhibitors (TSP-1, TIMP-1, and TIMP-2) in adipose tissues. The protein levels of VEGF, MMP-2 and MMP-9 were also decreased by ALS in adipose tissue. Metabolic changes in plasma lipids, liver triglycerides, and hepatic expression of fatty acid oxidation genes occurred during ALS-induced weight loss. These results suggest that ALS, which has antiangiogenic and MMP inhibitory activities, reduces adipose tissue mass in nutritionally obese mice, demonstrating that adipose tissue growth can be regulated by angiogenesis inhibitors.

## Introduction

The development of obesity is associated with extensive modifications of adipose tissue involving adipogenesis, angiogenesis and extracellular matrix (ECM) remodeling [1].

Similar to tumor growth, angiogenesis (the formation of new blood vessels from pre-existing vessels) occurs in growing adipose tissue of adults. Most tissues normally do not grow throughout adulthood and the supporting vasculature is quiescent, whereas adipose tissue can grow and regress throughout life. Adipose tissue is highly vascularized and each adipocyte is nourished by an extensive capillary network [1–3]. Extensive changes in ECM remodeling have also been observed during adipose tissue growth [4]. The matrix metalloproteinase (MMP) system plays an important role in the development of adipose tissue and microvessel maturation via ECM modulation [4–6]. Therefore, it has been suggested that adipose tissue growth is dependent upon angiogenesis and may be inhibited by angiogenesis inhibitors.

Actively growing adipocytes produce an array of vascular growth factors, including vascular endothelial growth factor (VEGF), fibroblast growth factor (FGF), soluble VEGF receptor-2 (VEGFR2), hepatocyte growth factor, angiopoietin-2 and angiogenin [7]. The serum concentrations of these growth factors are higher in overweight or obese individuals compared to normal-weight individuals [8]. In addition to secreting vascular growth factors, adipocytes release several MMPs that modulate the ECM and allow matrix-bound vascular growth factors to induce angiogenesis. Thus, angiogenesis and MMP inhibitors are in development as targeted antiobesity therapeutics [9].

We examined the antiangiogenic and MMP inhibitory activities of medicinal herbs and found that *Melissa officinalis* L. (Labiatae; lemon balm) exhibited antiangiogenic and MMP inhibitory activities [10]. *Melissa* has been used as a medicinal plant to treat nervousness, insomnia, gastrointestinal disorders, herpes virus infection and Alzheimer's disease [11,12]. An active fraction denominated ALS-L1023 (ALS) extracted from *Melissa* leaves by organic solvents exhibited antiangiogenic and MMP inhibitory activities. In this study, we investigated the effect of ALS on angiogenesis and MMP activities and whether ALS can regulate adipose tissue growth in high fat diet-induced obese mice. When high fat diet-induced obese mice were treated with ALS for 8 weeks, adipose tissue mass and adipocyte size were significantly reduced in treated mice compared to control mice. The mRNA expression of angiogenic factors (VEGF and bFGF), MMPs (MMP-2 and -9), and their inhibitors (TIMP-1, TIMP-2, and TSP-1) were also modulated by ALS in obese mice. Metabolic changes in circulating lipids, liver lipid accumulation, and hepatic expression of fatty acid oxidation-related genes were found during ALS-induced weight reduction. These studies suggest that ALS can inhibit the growth of adipose tissue by inhibiting angiogenesis and MMPs.

## Materials and Methods

### Preparation of ALS

*Melissa officinalis* L. leaves were purchased from Alfred Galke GmbH, (Harz, Germany) and ALS was manufactured by activity-guided fractionation. The dried *Melissa* leaves were extracted with aqueous ethanol and the extract was filtered and concentrated. The concentrated ethanol extract was further fractionated with ethyl acetate, concentrated and dried to obtain ALS in a dried powder form. ALS was standardized with two reference compounds of rosmarinic acid and caffeic acid by high-performance liquid chromatography (HPLC). ALS was dissolved in 100% DMSO and used for *in vitro* tests.

### *In vitro* Cytotoxicity Test

Human umbilical vein endothelial cells (HUVECs) were purchased from Lonza (Basel, Switzerland) and cultured in EBM-2 supplemented with SingleQuots (Lonza, Basel, Switzerland) in a 37°C incubator with a humidified atmosphere containing 5% CO_2_. HUVECs were plated on 96 well plate at a density of 1 × 10^4^ cells/well and incubated for 24 h at 37°C with culture medium in the absence or presence of 10, 25, 50, 75, 100 or 150 μg/ml ALS. Cell viability was detected by 2,3-bis[2-methoxy-4-nitro-5-sulfophenyl]-2H-tetrazolium-5-carboxanilide disodium salt (XTT) assay using a Cell Proliferation Kit II (Roche, Basel, Switzerland).

### HUVEC Proliferation Assay

To perform VEGF-induced or bFGF-induced HUVEC proliferation assay, HUVECs were cultured in EBM-2 supplemented with SingleQuots in a 37°C incubator with a humidified atmosphere containing 5% CO_2_. HUVECs were plated on 96-well plates at a density of 3 × 10^4^cells/well with EBM-2 medium containing 2% fetal bovine serum. After 24 hours, the cells were washed twice with phosphate-buffered saline (PBS) and then EBM-2 medium with or without 10 ng/ml of VEGF or bFGF was added to these cells in the absence or presence of 25 or 50 μg/ml of ALS. After 48 h, the proliferation of HUVECs was measured by the XTT test.

### MMP Assay

MMP activity was measured using an LS50B spectrofluorometer (Perkin-Elmer, Waltham, MA, USA) using the substrate 2,4-dinitrophenyl-Pro-Leu-Gly-Met-Trp-Ser-Arg (Calbiochem, San Diego, CA, USA), as previously described [13]. Recombinant human MMP-2 and MMP-9 were purchased from R&D Systems (Minneapolis, MN, USA) and used after activation with 1 mM APMA (amino-phenyl mercuric acetate) before the assay. MMP (10 nM) and substrate (1 μM) were mixed in 2 ml of reaction buffer (50 mM Tricine, pH 7.5, 10 mM CaCl_2_, 200 mM NaCl) in the presence or absence of ALS. Fluorescence intensity was measured at room temperature using a 280-nm excitation wavelength and a 360-nm emission wavelength.

### Animal Studies

Eight-week-old male wild-type C57BL/6J mice (n = 8/group) were housed and bred at the Mokwon University under pathogen-free conditions with a standard 12-h light/dark cycle. Prior to the administration of special diets, mice were fed standard rodent chow and water *ad libitum*. Mice were randomly divided into four groups, which were fed for 8 weeks with a standard chow diet (10% kcal fat, Research Diets, New Brunswick, NJ, USA), a high fat diet (45% kcal fat, Research Diets), or the same high fat diet supplemented with 0.4 or 0.8% (w/w) ALS. 4 or 8 g ALS-L1023 powder was mixed with 1 kg high fat diet. The body weight of each animal was measured daily by a person blinded to the treatments. Food intake was determined by measuring the amount of food consumed by the mice throughout the treatment period. A known amount of a diet was given to the animal. The food was reweighed every week and the amount consumed was calculated by difference. After a 12 h fast on the last day of the study, the animals were sacrificed by cervical dislocation [14]. Visceral (VSC) and subcutaneous (SC) fat pads were removed, weighed, snap-frozen in liquid nitrogen and stored at −80°C until use. Portions of the VSC and SC fat pads were prepared for histology. Plasma levels of triglycerides and free fatty acids were measured using an automatic blood chemical analyzer (CIBA Corning, Oberlin, OH) and SICDIA NEFAZYME (Shinyang Chemical, Seoul, Korea), respectively. All animal experiments were approved by the Institutional Animal Care and Use Committees of Mokwon University (permit number: NVRGS AEC-5) and conducted according to National Research Council Guidelines.

### Histological Analysis

For hematoxylin and eosin (H&E) staining, organs were fixed in 10% phosphate-buffered formalin for 1 day and processed in a routine manner for paraffin sections. Tissue sections (5 μm) were cut and stained with H&E for microscopic examination. To quantify the size of epididymal and inguinal adipocytes, the H&E-stained sections were analyzed using the Image-Pro Plus analysis system (Media cybernetics, Bethesda, MD). Blood vessel staining was performed using a blood vessel staining kit (Chemicon, Billerica, MA, USA). Paraffin sections (3-μm thick) of adipose tissues were incubated with a rabbit anti-von Willebrand Factor (vWF) antibody as a primary antibody, goat anti-rabbit antibody as a biotinylated secondary antibody and streptavidin-alkaline phosphatase solution. A freshly prepared chromogen reagent was added to sections for the visualization of blood vessel. Blood vessel density was determined by Image-Pro Plus analysis system and blood vessel density was normalized with the number of adipocytes.

### Zymography

MMP-2 and MMP-9 activities were monitored by zymography with extracts of epididymal and inguinal adipose tissues [15]. Adipose tissues were weighed and extracted with 10 mM sodium phosphate buffer (pH 7.2) containing 150 mM NaCl, 1% Triton X-100, 0.1% sodium dodecyl sulfate (SDS), 0.5% sodium deoxycholate and 0.2% NaN_3_ at 4°C. Adipose tissue extracts were mixed with zymography sample buffer (63 mM Tris HCl, 10% glycerol, 2% SDS, 0.0025% bromophenol blue, pH 6.8) without heat denaturation. The HT1080 cell culture medium was used for the molecular weight markers for MMP. Electrophoresis was performed at 125 V on 10% SDS-polyacrylamide gels containing 0.1% gelatin. After electrophoresis, the gels were incubated in renaturing buffer containing 0.25% Triton X-100 for 30 min at room temperature and equilibrated in developing buffer (50 mM Tris base, 40 mM HCl, 200 mM NaCl, 5 mM CaCl_2_, 0.2% Brij-35) for 30 min at room temperature. The gels were then incubated in developing buffer overnight at 37°C. The gels were stained with 0.1% Coomassie Blue R-250 and destained with 10% acetic acid in 40% methanol.

### Reverse Transcription-Polymerase Chain Reaction (RT-PCR)

Total cellular RNA from retroperitoneal and inguinal adipose tissues was prepared using Trizol reagent (Gibco-BRL, Grand Island, NY, USA). After 2 μg total RNA was reverse-transcribed using Moloney murine leukemia virus reverse transcriptase and an antisense primer, cDNA was generated. Synthesized cDNA fragments were amplified by PCR in an MJ Research Thermocycler (Waltham, MA, USA). The PCR primers used for gene expression analysis are shown in S1 Table. The cDNA was mixed with PCR primers, *Taq* DNA polymerase (Nanohelix, Daejeon, Korea), and a deoxyribonucleotide triphosphate mixture. The reaction consisted of 30 cycles of denaturation for 1 min at 94°C, annealing for 1 min at 58°C and elongation for 1 min at 72°C. PCR products were quantified from agarose gels using GeneGenius (Syngene, Cambridge, UK).

### Western Blot Analysis

Epididymal and inguinal adipose tissues were lysed in ice-cold lysis buffer (50 mM Tris-Cl (pH 8.0), 150 mM NaCl, 0.02% Sodium azide and 1% Triton X-100) containing protease inhibitors (phenylmethylsulfonyl fluoride and aprotinin). Lysates were centrifuged at 12,000 rpm for 20 min at 4°C and the resulting supernatants (10 μg) were subjected to electrophoresis on 10% polyacrylamide gels. The separated proteins were transferred to PVDF membrane (Millipore, Billerica, MA, USA). Membranes were incubated with primary antibodies from Santa Cruz Biotechnology (Santa Cruz, CA, USA). The primary antibodies were anti-VEGF antibody (sc-507), anti-MMP-2 antibody (sc-10736) and anti-MMP-9 antibody (sc-10737) (1:200 dilution). After incubating with HRP-conjugated goat anti-rabbit IgG (sc-2004, Santa Cruz) (1:2500 dilution) as a secondary antibody, blots were visualized using an ECL Western blot detection system (Intron, Daejeon, Korea).

### Statistics

The data distribution was analyzed for normality and the group means and standard deviation (SD) were measured. All values were expressed as mean ± SD. Statistical analysis was performed by ANOVA followed by the post hoc Tukey’s multiple comparison test to analyze which group means differ and how many group means differ from each other. Statistical significance was defined as a value of *p*<0.05.

## Results

### Inhibition of Angiogenesis and MMP Activity by ALS

**Fig 1:**
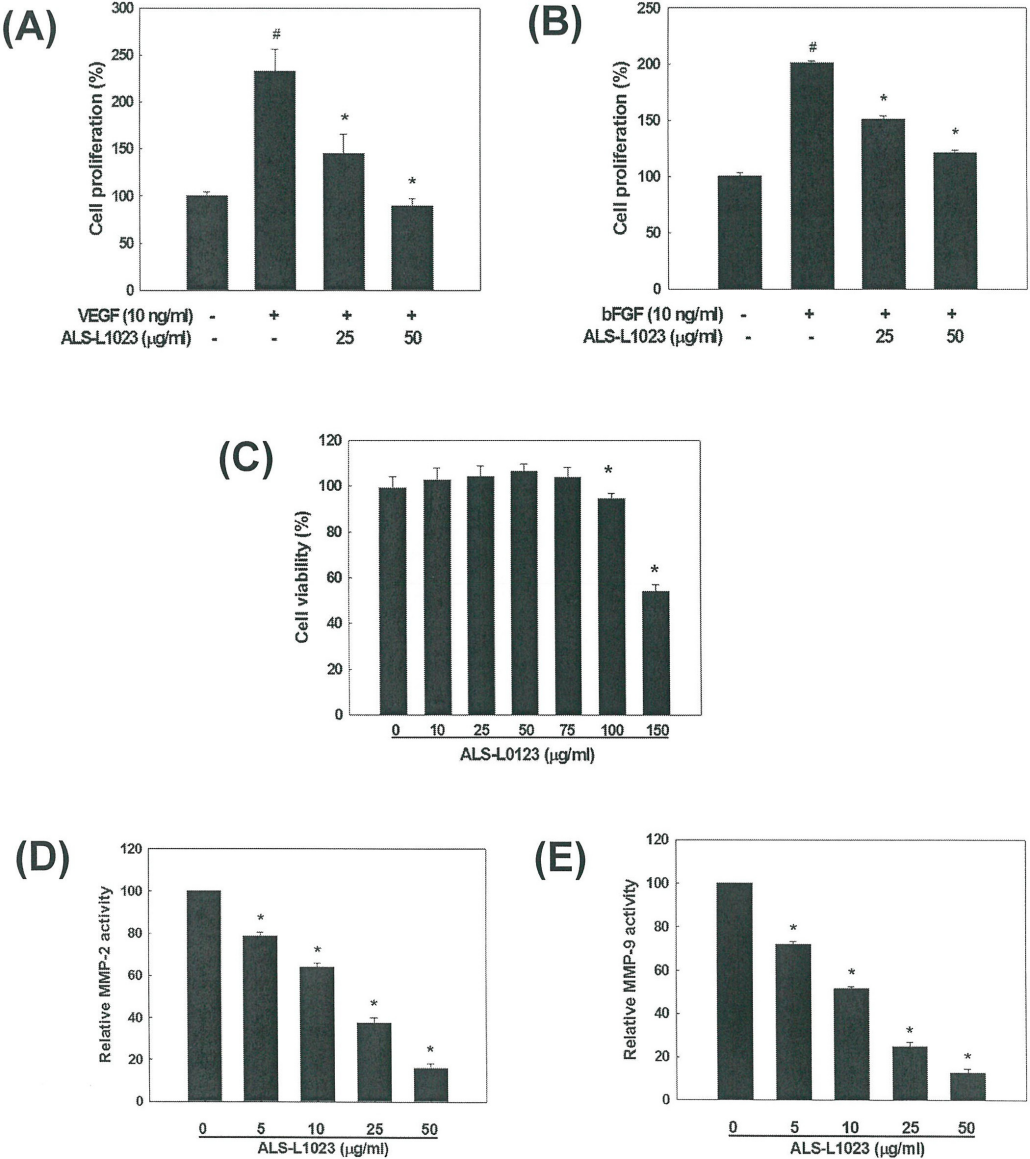
Inhibitory effects of ALS on angiogenesis and MMP activity. (A) Inhibition of VEGF-induced HUVEC proliferation by ALS. (B) Inhibition of bFGF-induced HUVEC proliferation by ALS. #p<0.05 compared to vehicle control, *p<0.05 compared to VEGF- or bFGF-treated control group. (C) The effect of ALS on HUVEC cell viability by MTT assay. * p<0.05 compared to the vehicle control. ALS-mediated inhibition of (D) MMP-2 and (E) MMP-9 activity measured by spectrofluorometry and the IC_50_ values were determined. * p<0.05 compared to the vehicle control.

The effect of ALS on angiogenesis was examined using HUVEC proliferation assays. VEGF and bFGF are angiogenic stimulators that play important roles in pathogenic angiogenesis by inducing endothelial cell proliferation. ALS added to the culture medium at a concentration of 25 μg/ml or 50 μg/ml inhibited VEGF-induced endothelial cell proliferation by 68% and 100%, respectively, compared to control (p<0.05; Fig 1A). ALS also inhibited bFGF-induced HUVEC proliferation by 48% and 86% at a concentration of 25 μg/ml and 50 μg/ml, respectively, compared to control (p<0.05; Fig 1B). Inhibition of endothelial cell proliferation by ALS was not due to cytotoxic effect since the viability of HUVECs was not affected by ALS at concentrations between 10 μg/ml and 75 μg/ml determined by XTT assays (Fig 1C). Thus, the antiangiogenic effects of ALS were mediated by inhibition of VEGF-induced or bFGF-induced endothelial cell proliferation.

MMP-2 and MMP-9 contribute to tissue remodeling by degrading the ECM in angiogenic processes as well as participate in adipocyte differentiation [6]. The activities of MMP-2 and MMP-9 were significantly inhibited by ALS treatment in a concentration-dependent manner (p<0.05; Fig 1D and 1E). The IC_50_ values were 17.7±1.0 μg/ml for MMP-2 and 12.3±1.4 μg/ml for MMP-9. Thus, ALS possesses inhibitory effects on both angiogenesis and MMP activity.

### Effects of ALS on Body Weight and Adipose Tissue Mass

**Fig 2:**
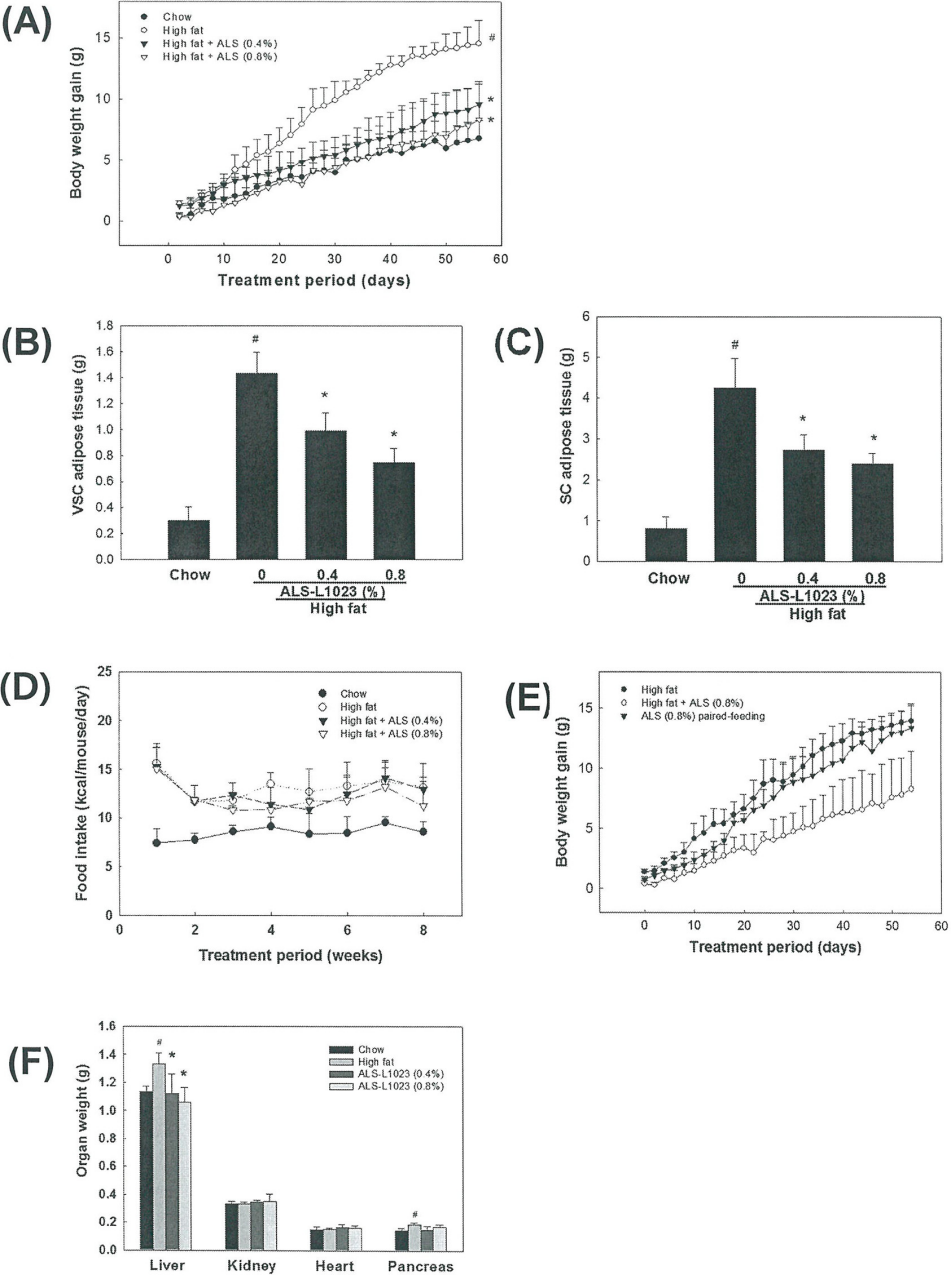
Regulation of body weight gain, adipose tissue mass and food consumption in high fat diet-fed obese mice. Adult male mice were fed a standard chow diet, a high fat diet, or high fat diet supplemented with 0.4 or 0.8% ALS for 8 weeks. (A) Modulation of body weight gain by ALS. Body weight gains at the end of the treatment period are significantly different between the chow diet group and the high fat diet group (#p<0.05) and between the high fat diet group and the groups fed a high fat diet supplemented with 0.4 or 0.8% ALS (*p<0.05). Regulation of VSC (B) and SC (C) fat mass by ALS. (D) Effects of ALS on food intake. (E) Appetite of ALS-treated mice. We fed the amount consumed per day by each treated mouse to a paired mouse and measured body weights daily. (F) Organ weights for liver, kidney, heart and pancreas. All values are expressed as the mean ± SD. #p<0.05 compared to the chow group, *p<0.05 compared to the high fat group.

To determine whether ALS reduces adipose tissue mass and prevents body weight gain in obese mice, mice were fed a low fat diet, a high fat diet or the same high fat diet supplemented with 0.4% or 0.8% ALS for 8 weeks. After 8 weeks of treatment, the high fat diet-fed mice had 115% greater body weight gains compared to that of standard chow diet-fed mice (14.56±1.89 g vs. 6.78±1.45 g, respectively, p<0.05). In contrast, mice fed a high fat diet supplemented with 0.4% and 0.8% ALS had 34% (9.56±1.69 g) and 43% (8.32±3.13 g) lower body weights, respectively, compared to obese mice fed a high fat diet alone (p<0.05; Fig 2A). Adipose tissue mass was significantly decreased by ALS treatment in high fat diet-fed obese mice. As shown in Fig 2B and 2C, the mass of both VSC and SC fat pads in the ALS-treated mice was reduced in comparison to that of high fat diet-fed control mice. Both VSC and SC adipose tissue weights were decreased by 31% (p<0.05) and 36% (p<0.05) after 0.4% ALS and 48% (p<0.05) and 44% (p<0.05) after 0.8% ALS administration, respectively.

Mice fed a high fat diet had higher food consumption compared to mice fed a low fat diet, but there was not a significant difference in food consumption between the high fat diet control group and ALS-treated group (Fig 2D). In pair feeding experiments, 0.8% ALS had no significant appetite effect (Fig 2E). ALS treatment had no effect on organ weight of the kidney and heart showing that ALS is targeting only growing adipose tissue, whereas the liver weight increased by high fat diet was decreased to weight of chow-fed mice after ALS treatment (Fig 2F).

### Effects of ALS on Adipocyte Size

**Fig 3:**
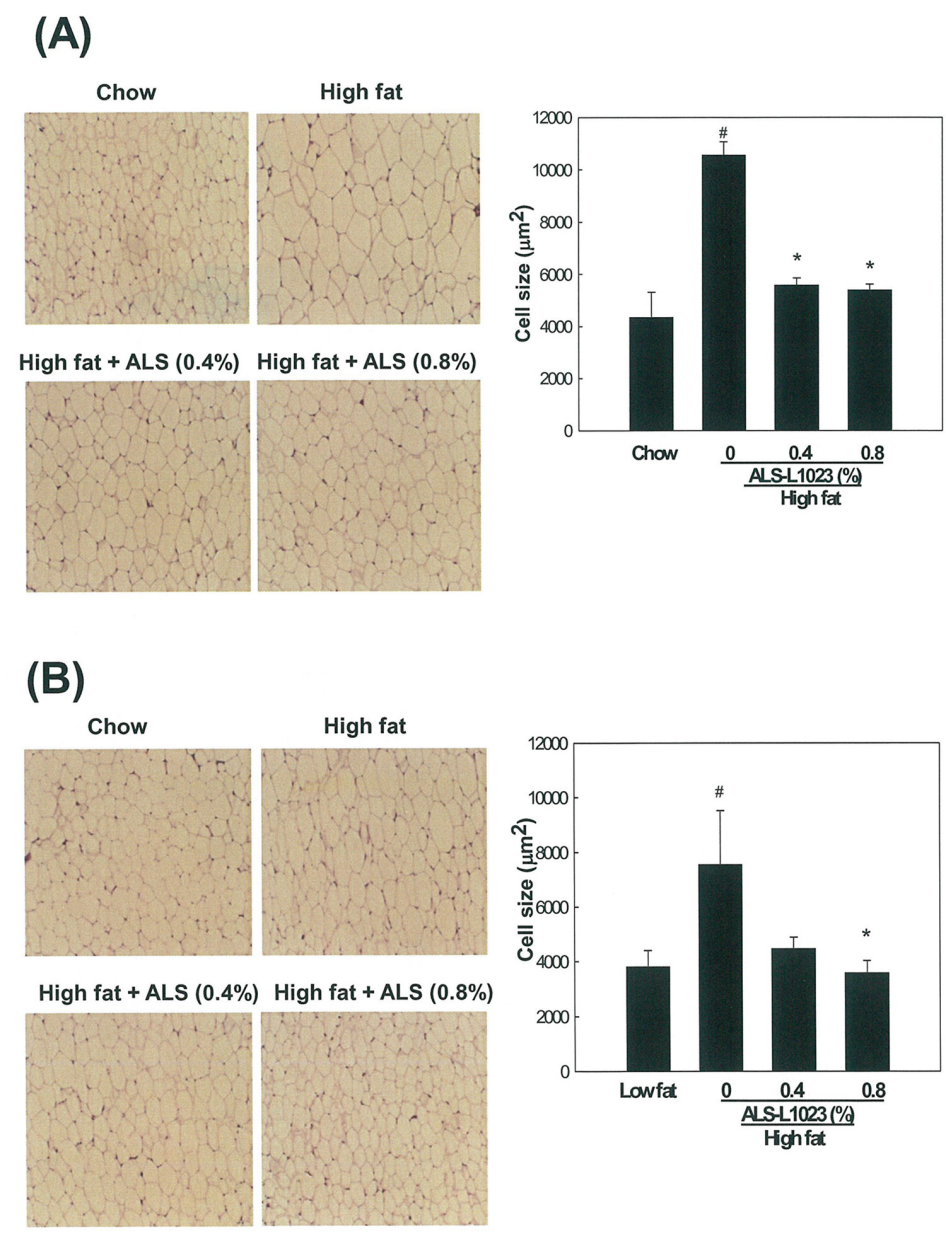
Light microscopy analysis of adipocyte size in adipose tissue. Adult male mice were fed a standard chow diet, a high fat diet, or the same high fat diet supplemented with 0.4 or 0.8% ALS for 8 weeks. Representative H&E-stained sections (5-μm thick) of (A) epididymal VSC and (B) inguinal SC adipose tissues are shown (original magnification ×100). Adipocyte size in the high fat diet plus ALS groups was smaller compared to the high fat diet groups. The size of adipocytes in a fixed area (1,000,000 μm^2^) was measured. All values are expressed as the mean ± SD. #p<0.05 compared to the chow group, *p<0.05 compared to the high fat group.

Analysis of H&E-stained adipose tissue sections showed that ALS treatment significantly decreased the size of adipocytes. ALS at concentrations of 0.4% and 0.8% decreased the average size of VSC adipocytes by 47% and 48%, respectively (p<0.05; Fig 3A). The average size of adipocytes was 5571±271 μm^2^ in 0.4% ALS-treated mice and 5394±216 μm^2^ in 0.8% ALS-treated mice, which were smaller compared to the high fat diet-fed obese mice (10565±495 μm^2^). ALS at concentrations of 0.4% and 0.8% also decreased the average size of SC adipocytes by 40% and 52%, respectively (p<0.05; Fig 3B). The average size of adipocytes was 4475±420 μm^2^ in 0.4% ALS-treated mice and 3585±446 μm^2^ in 0.8% ALS-treated mice, which were smaller compared to the high fat diet-fed mice (7544±1978 μm^2^).

### Effects of ALS on Vascularization in Adipose Tissue

**Fig 4:**
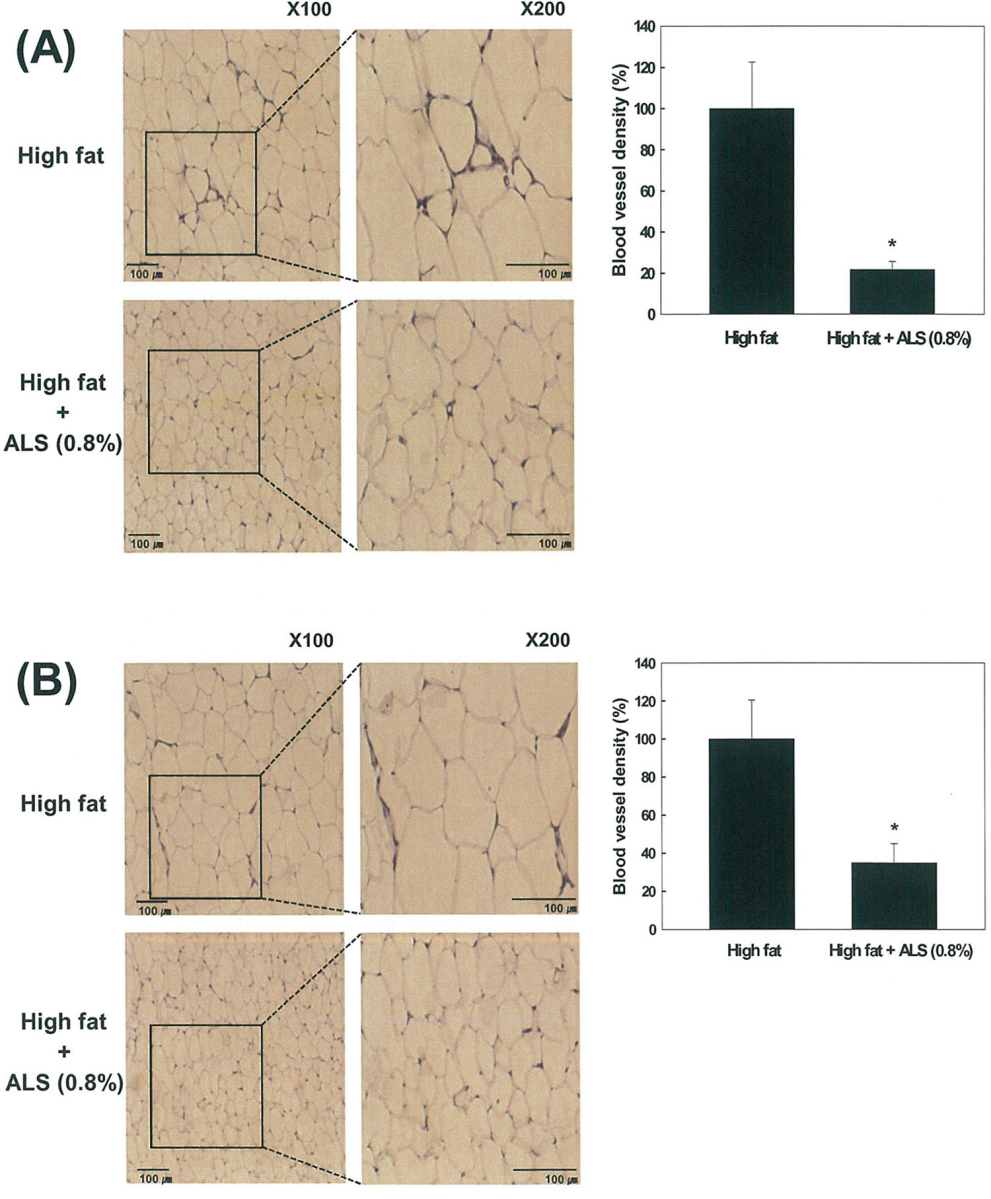
Histological analysis of blood vessels in adipose tissue stained with an antibody against vWF. The blood vessels of (A) epididymal VSC and (B) inguinal SC adipose tissues derived from mice fed a high fat diet or a high fat diet supplemented with 0.8% ALS for 8 weeks were stained and analyzed (original magnification ×100). Higher magnitude of bracket area is shown (original magnification ×200). All values are expressed as the mean ± SD. *p<0.05 compared to the high fat group.

To determine whether the decrease of adipose tissue mass by ALS treatment resulted from the inhibition of angiogenesis, we studied the effects of ALS on blood vessel density in both VSC and SC adipose tissue. Staining of adipose tissue sections with an antibody against vWF, an endothelial cell marker showed that the blood vessel density of both VSC and SC adipose tissue from the ALS-treated mice was significantly lower compared to the high fat diet-fed control mice. ALS treatment decreased blood vessel density by 78% in VSC adipose tissue (p<0.05; Fig 4A) and by 65% in SC adipose tissue (p<0.05; Fig 4B).

### Effects of ALS on MMP Activity in Adipose Tissue

**Fig 5:**
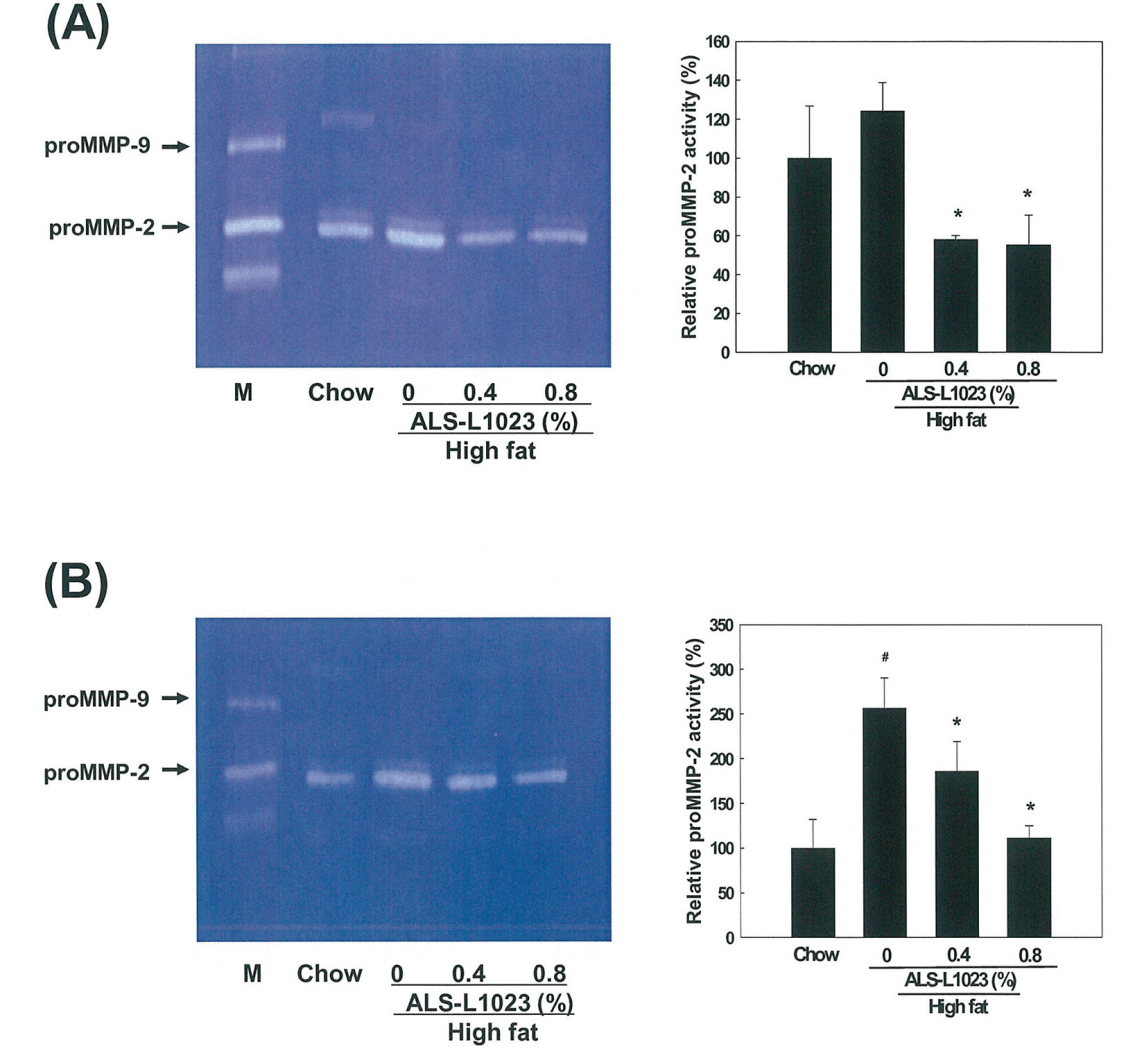
Zymographic analysis of adipose tissue. Extracts from (A) epididymal VSC and (B) inguinal SC adipose tissues obtained from mice fed a high fat diet or a high fat diet supplemented with 0.4 and 0.8% ALS for 8 weeks were applied to a gelatin-containing gel. Gelatinolytic activity was measured by zymography. The HT1080 cell culture medium was used for the molecular weight markers for MMP-2 and MMP-9. All values are expressed as the mean ± SD. #p<0.05 compared to the chow group, *p<0.05 compared to the high fat group. M, molecular weight marker.

MMP activity in adipose tissue extracts was examined using zymography on gelatin-containing gels. Gelatin zymography showed that proMMP-2 activity was significantly reduced in the adipose tissue from the ALS-treated mice compared to the control group, whereas proMMP-9 levels were not detectable. The proMMP-2 activity in VSC adipose tissue was significantly reduced by 48% and 49% after the administration of 0.4% and 0.8% ALS, respectively (p<0.05; Fig 5A). Similarly, proMMP-2 activity in SC adipose tissue was also decreased by 23% and 36% after treatment with 0.4% and 0.8% ALS, respectively (p<0.05; Fig 5B). Thus ALS reduced proMMP-2 activity more in VSC adipose tissue than in SC adipose tissue.

### Effects of ALS on mRNA and Protein Expression of Angiogenic Factors, MMPs, and Their Inhibitors in Adipose Tissue

**Fig 6:**
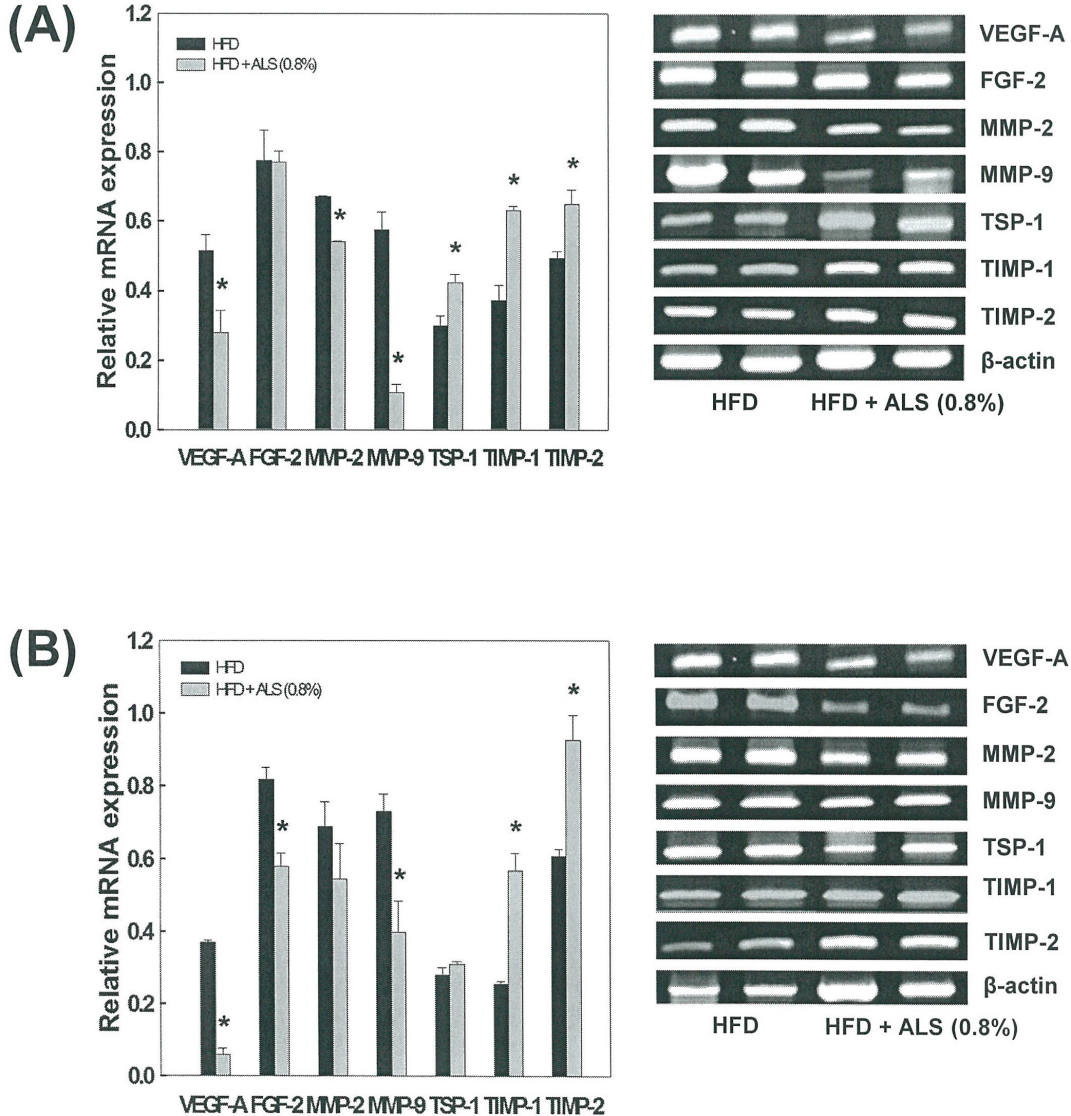
Effects of ALS on the expression of angiogenic factors, MMPs and their inhibitors in epididymal VSC and inguinal SC adipose tissues of diet-induced obese mice. Adult male mice were fed a high fat diet or a high fat diet supplemented with 0.8% ALS for 8 weeks. Analysis of VEGF-A, FGF-2, MMP-2, MMP-9, TIMP-1, TIMP-2 and TSP-1 mRNA levels by RT-PCR in epididymal VSC (A) and inguinal SC (B) adipose tissues. Representative PCR bands from one of three independent experiments are shown. All values are expressed as the mean ± SD. *p<0.05 compared to the high fat group.

The expression patterns of genes involved in angiogenesis were investigated in VSC and SC adipose tissues from C57BL/6J mice fed a high fat diet. The mRNA expression of angiogenic and antiangiogenic factors was downregulated and upregulated, respectively, in ALS-treated mice compared to high fat diet-fed control mice. In VSC adipose tissue, the mRNA expression of the VEGF-A angiogenic factor was decreased by 45% (p<0.05) compared to control mice, whereas the mRNA level of the TSP-1 antiangiogenic factor was increased by 41% in ALS-treated mice compared to control mice (p<0.05; Fig 6A). In SC adipose tissue, the mRNA levels of VEGF-A and FGF-2 were significantly reduced by 83% (p<0.05) and 29% (p<0.05), respectively, whereas TSP-1 mRNA level was increased by 11% in ALS-treated mice compared to control mice (Fig 6B).

MMP mRNA expression was significantly inhibited by ALS treatment. In VSC adipose tissue, MMP-2 and MMP-9 mRNA levels were significantly decreased by 19% (p<0.05) and 81% (p<0.05), respectively (Fig 6A), and in SC adipose tissue significantly decreased by 20% and 41% (p<0.05), respectively (Fig 6B). In contrast, TIMP-1 and TIMP-2 mRNA levels were significantly increased by 69% (p<0.05) and 31% (p<0.05), respectively, in VSC adipose tissue (Fig 6A) and significantly increased by 122% (p<0.05) and 43% (p<0.05), respectively, in SC adipose tissue (Fig 6B) from ALS-treated mice compared to control mice.

**Fig 7:**
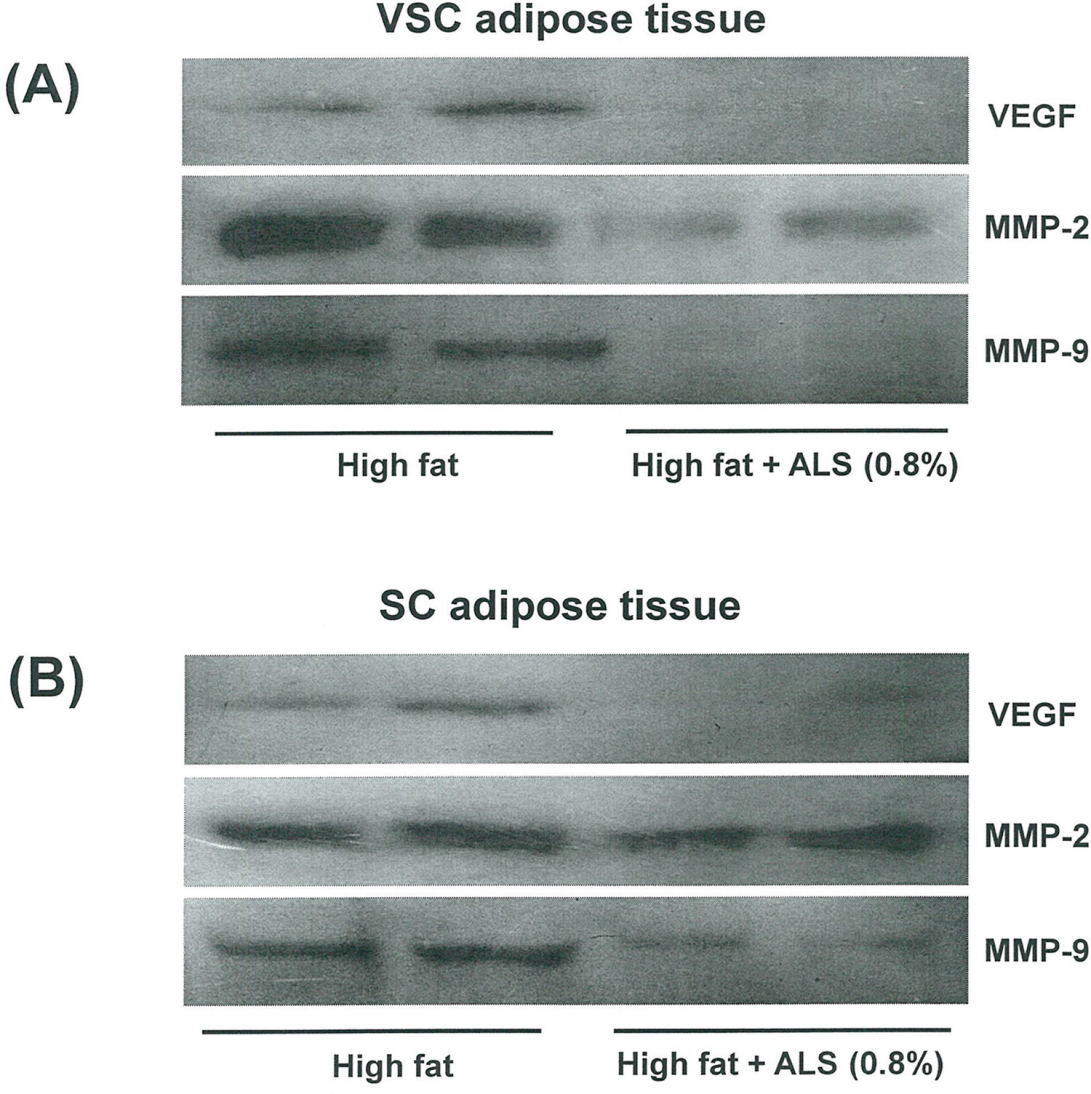
Effects of ALS on the protein expression of VEGF, MMP-2 and MMP-9 in epididymal VSC and inguinal SC adipose tissues of diet-induced obese mice. The protein levels of epididymal VSC and inguinal SC adipose tissues derived from mice fed a high fat diet or a high fat diet supplemented with 0.8% ALS for 8 weeks were analyzed by Western blotting. Representative bands from one of three independent experiments are shown.

Consistent with the result of mRNA expression, the protein level of the VEGF angiogenic factor was decreased in ALS-treated mice compared to high fat diet-fed control mice in VSC and SC adipose tissue. Similar to VEGF, the protein levels of MMP-2 and MMP-9 were decreased in ALS-treated mice compared to high fat diet-fed control mice in both VSC and SC adipose tissue (Fig 7).

### Effects of ALS on Plasma Lipid Levels, Hepatic Lipid Accumulation, and mRNA Expression of Hepatic Fatty Acid Oxidation-Related Genes

**Fig 8:**
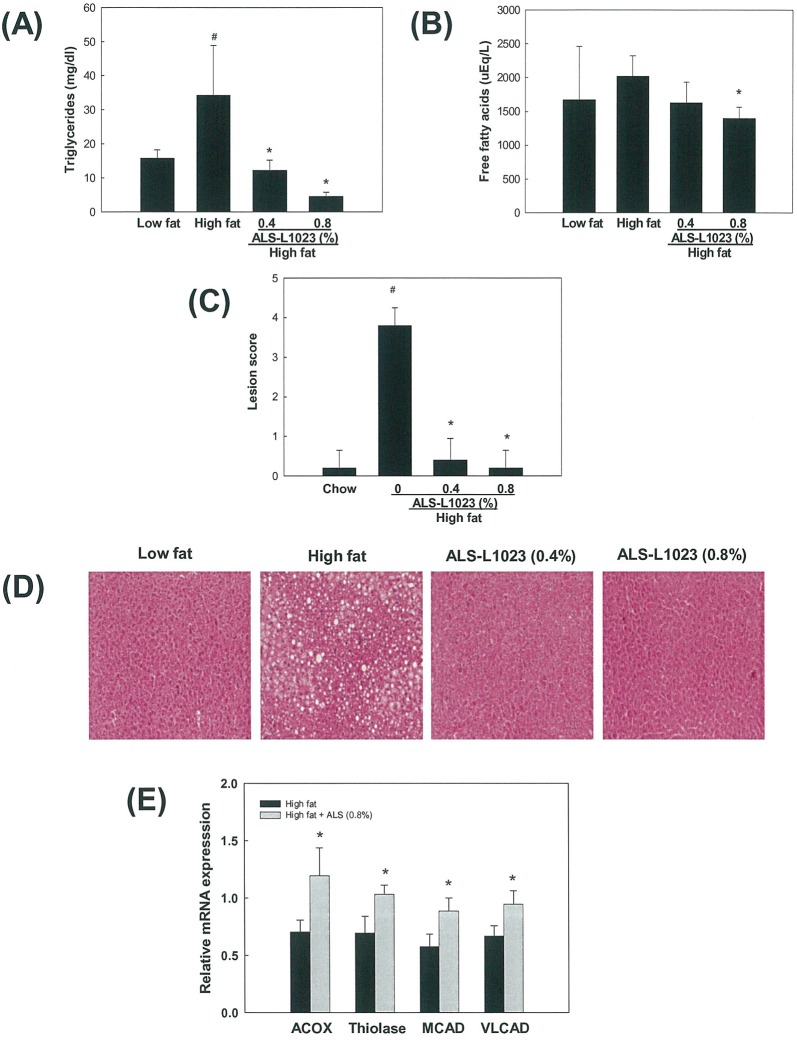
Effects of ALS on plasma lipid levels, hepatic lipid accumulation and liver PPARα target gene expression in diet-induced obese mice. Adult male mice were fed a high fat diet or a high fat diet supplemented with 0.8% ALS for 8 weeks. Plasma levels of (A) triglycerides and (B) free fatty acids. (C) Histological analyses of hepatic lipid accumulation. Pathological scores of hepatic lipid accumulation are as follows: 0, no lesion; 1, mild; 2, moderate; 4, very severe. (D) Representative H&E-stained liver sections are shown (original magnification ×100). (E) Modulation of liver PPARα target gene expression. All values are expressed as the mean ± SD. *p<0.05 compared to the high fat group.

Circulating levels of triglycerides and free fatty acids were higher by 117% and 21%, respectively, in high fat diet-fed mice compared with chow diet-fed mice (Fig 8A and 8B). However, triglyceride and free fatty acid levels were decreased by 87% and 31%, respectively, in 0.8% ALS-L1023 treated mice compared with those in high fat diet-fed mice. Significant hepatic lipid accumulation was induced by high fat, as indicated by the increased hepatic lesion score (Fig 8C and 8D). In contrast, ALS-L1023 almost completely abolished hepatic triglycerides. We then determined the levels of mRNA encoding peroxisome proliferator-activated receptor α (PPARα) target enzymes responsible for fatty acid β-oxidation in the livers. Compared with high fat group, the mRNA levels of two peroxisomal PPARα targets, namely acyl-CoA oxidase (ACOX) and thiolase, were increased in 0.8% ALS-treated group by 54% and 49%, respectively (Fig 8E). mRNA encoding mitochondrial PPARα targets, medium chain acyl-CoA dehydrogenase (MCAD) and very long chain acyl-CoA dehydrogenase (VLCAD), were also elevated by 54% and 40%, respectively.

## Discussion

Angiogenesis, the formation of new capillary blood vessels, is a tightly regulated process. Under normal physiological conditions, angiogenesis only takes place during embryonic development, wound healing and female menstruation [16]. Failure in the regulation of angiogenesis is correlated with many diseases such as cancer, rheumatoid arthritis, psoriasis and proliferative retinopathy [17, 18]. Similarly, the growth and expansion of adipose tissue requires the formation of new blood vessels to provide oxygen and nutrients to adipocytes. Recently, we demonstrated that the antiangiogenic dietary supplement, Ob-X which is composed of a standardized mixture of *Melissa officinalis* L., *Morus alba* L. and *Artemisia capillaris* Thunb. aqueous extract reduces adipose tissue mass and suppresses obesity by inhibiting angiogenesis in nutritionally and genetically obese mice [14, 19].

Since one of the Ob-X ingredients, aqueous extract of *Melissa* leaves showed anti-angiogenic effects we extracted *Melissa* leaves with organic solvents in an attempt to increase anti-angiogenic activity. In the present study we prepared ALS by a two-step organic solvent fractionation from *Melissa* leaves which was standardized with reference compounds by HPLC. The antiangiogenic and MMP inhibitory activities of ALS were enhanced compared to the water extract or aqueous ethanol extract of *Melissa* leaves suggesting that hydrophobic active components in ALS extracted with organic solvents may have more potent antiangiogenic effects compared to the hyrophilic active components extracted with water or aqueous ethanol (data not shown).

We examined the effects of ALS on angiogenesis and MMP activity *in vitro*. ALS reduced VEGF- and bFGF-induced HUVEC proliferation, demonstrating that ALS exerts antiangiogenic effects by inhibiting the proliferation of endothelial cells. ALS also inhibited the activities of two major MMPs (MMP-2 and MMP-9) in a concentration-dependent manner *in vitro*. Since it was suggested that inhibition of angiogenesis and MMP activity impairs adipose tissue development [4, 20], it is likely that ALS may suppress adipose tissue growth by inhibiting angiogenesis and MMPs.

We then treated high fat diet-induced obese mice with ALS. Consistent with our hypothesis that ALS can inhibit adipose tissue growth, ALS substantially reduced adipose tissue mass and prevented body weight gain. We previously had performed animal experiments using ALS at doses of 0.1, 0.25, 0.5% in high fat diet-induced obese rats and found that 0.5% ALS (100mg/kg/day) significantly reduced adipose tissue mass (results not published yet). Considering dosage conversion from rat to mouse, we selected ALS at doses of 0.4% (200mg/kg/day) and 0.8% (400mg/kg/day) to supplement the HFD in an obese mouse model to increase the effects for a mechanism study. Treatment with 0.4% and 0.8% ALS for 8 weeks decreased body weight gain by 70% and 84%, respectively. Both VSC and SC adipose tissue weights were decreased by 65% (p<0.05) and 68% (p<0.05) after 0.4% ALS and 74% (p<0.05) and 72% (p<0.05) after 0.8% ALS administration, respectively. The ALS-mediated decreases in VSC and SC adipose tissue mass were greater compared to reductions in VSC (59%) and SC (65%) adipose tissue mass obtained after a 12-week administration of galardin, a broad spectrum inhibitor of MMPs and angiogenesis [4]. Our data are also supported by other results which indicate that several types of angiogenesis inhibitors, such as angiostatin, endostatin and TNP-470 and its analog CKD-732 inhibit fat mass expansion in mice [20–22]. Similar to the effects of ALS on adipose tissue mass, the average size of adipocytes in both VSC and SC adipose tissues was substantially smaller in ALS-treated mice than in untreated obese mice. These results indicate that ALS can reduce adipose tissue mass and inhibit adipocyte hypertrophy. VSC obesity is known to be closely associated with metabolic syndromes including insulin resistance [23, 24]. Given that adipogenesis and obesity are closely associated with angiogenesis, modulating angiogenesis may be a novel therapeutic approach to obesity and obesity-related diseases [25].

During ALS-induced adipose tissue loss, food intake was not changed, showing that ALS did not affect appetite. Furthermore, ALS-treated mice weighed less than pair-fed mice having similar calorie intake. Similarly, it was reported that the administration of angiogenesis inhibitors, such as endostatin, to *ob/ob* mice resulted in weight loss without changes in appetite [20] and tolylsam, an MMP inhibitor with specificity for gelatinases, reduced body weight and fat pad weight without an effect on food intake [26]. ALS may target adipose tissue and cause weight reduction. The weight loss induced by antiangiogenic ALS arose specifically from the loss of adipose tissue mass, as the weights of other organs such as the heart and kidneys were not reduced in ALS-treated obese mice. However, the liver weight increased by high fat diet was decreased to weight of chow-fed mice after ALS treatment. Thus, ALS can reduce adipose tissue mass by targeting only the growing adipose tissue without any side effects on other organs.

Blood vessel staining showed that the blood vessel density of both VSC and SC adipose tissues was reduced in ALS-treated mice compared to untreated obese mice. These data were supported by our previous results which showed that the antiangiogenic herbal composition Ob-X decreased blood vessel density of VSC adipose tissue in nutritionally obese mice [14]. Our present results suggest that ALS is a potent angiogenesis inhibitor and regulates adipose tissue mass by inhibiting angiogenesis.

As angiogenesis may represent a target for treating obesity, it is important to determine the expression profiles of genes involved in angiogenesis. Growing adipose tissue contains a diverse population of cell, which determines the expression of several angiogenic modulators. Angiogenic factors, such as VEGFs and FGF-2, promote the proliferation, differentiation and migration of endothelial cells within fat tissue [27, 28] Moreover, VEGF-A and FGF-2 synergistically induce angiogenesis [29]. Blockage of the VEGFR2 signaling system by a neutralizing antibody inhibits both angiogenesis and preadipocyte differentiation, suggesting that VEGFs act on endothelial cells to regulate preadipocyte differentiation [30]. In contrast, TSP-1 inhibits angiogenesis *in vivo* and impairs the migration and proliferation of cultured microvascular endothelial cells [31]. Our RT-PCR analysis showed that ALS administration to obese mice decreased VEGF-A and FGF-2 mRNA expression, whereas the antiangiogenic TSP-1 mRNA expression was increased in both VSC and SC adipose tissues. Western blot analysis confirmed that ALS treatment decreased VEGF protein level in VSC and SC adipose tissues.

In obesity, MMP expression is modulated in adipose tissue and MMPs (e.g., MMP-2 and MMP-9) potentially affect adipocyte differentiation. It has been reported that the different MMPs do not present the same behavior in all the obesity models. Maquoi et al [32] showed that the expression of MMP-3, -11, -12, -13 and -14 and TIMP-1 mRNA was upregulated, whereas that of MMP-7, -9, -16, and -24 and TIMP-4 was downregulated in high fat diet-induced obese mice. Similar results were reported in two genetic models of obesity (ob/ob and db/db mice) and in a diet-induced model of obesity [33]. In contrast to the data of Maquoi et al, Chavey et al found that mRNA levels for MMP-2, -3, -12, -14, -19 and TIMP-1 were strongly induced in obese adipose tissue, but MMP-7 and TIMP-3 mRNAs are markedly decreased in obesity. Other study demonstrated that MMP-2 and MMP-9 activity in VSC adipose tissue was decreased in an animal model of early insulin resistance [34]. However, this is not animal model of obesity and there was no difference in body weight compared with control mice. High MMP-2 expression was observed in the adipose tissue of mice with nutritionally induced obesity as well as in genetically obese mice [35]. Treatment with MMP inhibitors impairs adipose tissue development in mice fed a high fat diet [4, 36]. Furthermore, the secretion of MMP-2 and MMP-9 increases during adipocyte differentiation in both human adipocytes and mouse preadipocyte cell lines [6, 33], suggesting that MMP-2 and MMP-9 are important for adipocyte development. In this study, treating obese mice with ALS decreased MMP-2 and MMP-9 protein levels in adipose tissues. ALS also reduced MMP-2 and MMP-9 mRNA expression and increased TIMP-1 and TIMP-2 mRNA expression in both VSC and SC adipose tissues, indicating that ALS exerts a specific regulatory effect on genes and consequently proteins involved in angiogenesis and the MMP system in adipose tissues. Our observations further indicate that the inhibition of adipose tissue growth by angiogenesis inhibitors may alter the expression of genes responsible for angiogenesis and the MMP system.

Gelatin zymography of VSC and SC adipose tissue extracts showed that ALS reduced proMMP-2 activity compared to the control, although proMMP-9 activity was not detectable. It has been reported that, in galardin-treated animals, zymography on gelatin-containing gels showed reduced MMP-2 activity in SC and VSC adipose tissues, whereas MMP-9 level were not observed [4].

MMPs and TIMP expression profiles were profoundly altered in adipose tissue of high fat diet-induced obese mice. Most of these modulations were specific to the gonadal VSC fat, not to the SC fat [16]. In our study, gelatin zymography showed that MMP activity in VSC adipose tissue was more significantly reduced by ALS than in SC adipose tissue. However, there is no difference in reduction of adipose tissue mass between VSC and SC fat. The effect of ALS on reduction of adipose tissue mass might arise from anti-angiogenic activity as well as MMPs inhibitory activities.

Treatment with MMP inhibitors impairs adipose tissue development in mice fed a high fat diet [36]. Indeed, MMP-2^−/−^ mice had reduced fat mass and smaller adipocyte size in both VSC and SC adipose tissue compared to MMP-2^+/+^ mice, suggesting a functional role of MMP-2 in adipose tissue growth [37]. Thus, these data demonstrate that the inhibition of MMP activity by ALS may lead to reduced adipose tissue mass in obese mice.

It was reported that MMPs play important roles in angiogenesis and MMP-2 and MMP-9 activities indirectly facilitate angiogenesis whereas MMP inhibitors, both synthetic and endogenous, inhibit angiogenic responses both *in vivo* and *in vitro* [38, 39]. Moreover, MMP-deficient mice exhibit delayed or diminished angiogenic responses during development or in response to tumor xenograft [40]. On the other hand, it was also reported that MMP-based proteolysis of ECM proteins releases anti-angiogenic cryptic fragments such as angiostatin and endostatin [41, 42], showing that inhibiting MMP activity may decrease endogenous angiogenic inhibitors. However, studies demonstrated that MMPs could increase the bioavailability of potent angiogenic factors by releasing matrix-bound VEGF and basic FGF (bFGF) [43, 44]. Thus, suppressing MMP activity may decrease endogenous anti-angiogenic substances, whereas it decreases potent angiogenic factors such as VEGF and bFGF. In addition, MMPs have novel function of modulating adipocyte differentiation, which is independent of angiogenesis. Therefore, MMP inhibitors can block the adipocyte differentiation process [6, 33, 45]. Collectively, it seems that MMPs and their inhibitors play a pivotal role in controlling adipogenesis and adipose tissue growth.

Metabolic changes including plasma lipids, liver triglycerides, and hepatic expression of fatty acid oxidation-related genes occurred during ALS-induced weight loss. ALS treatment not only decreased circulating triglycerides and free fatty acids, but also inhibited liver lipid accumulation. Consistently, ALS-treated mice had significantly higher mRNA levels of hepatic PPARα target enzymes involved in fatty acid β-oxidation, indicating that the elevated fatty acid oxidation in the livers may be paralleled by large reductions in plasma lipids and weight gain. It is well known that PPARα increases the fatty acid catabolism by stimulating the transcription of rate-limiting enzymes in mitochondrial and peroxisomal β-oxidation and microsomal ω-oxidation [46]. It is likely that ALS stimulates the transcriptional expression of PPARα target enzymes in the livers, which reduces the intracellular levels of fatty acids available for triglyceride synthesis. This in turn curbs circulating triglyceride levels and fat accumulation. Thus, ALS may play a critical role in the regulation of obesity-associated disorders, such as hypertriglyceridemia, hepatic steatosis, insulin resistance and type 2 diabetes.

In conclusion, our present results demonstrate that ALS, which inhibits angiogenesis and MMP activity, suppresses the growth and development of adipose tissue in obese mice. These events may be mediated by changes in the expression of genes involved in angiogenesis and the MMP system. Thus, antiangiogenic ALS provides a potential therapeutic approach for preventing and treating human obesity and its related disorders.

## Supporting Information

S1 Table Sequences of primers used for RT-PCR assays. (DOC)
