# Supplementary material for: Reduction of Adipose Tissue Mass by the Angiogenesis Inhibitor ALS-L1023 from Melissa officinalis
Source: PLoS One. 2015 Nov 23;10(11):e0141612. doi: 10.1371/journal.pone.0141612 (PMC4658083; doi:10.1371/journal.pone.0141612)
Supplement: S1 Table — (DOC) [file pone.0141612.s001.doc]

**S1 Table. Sequences of primers used for RT-PCR assays**

| **Gene** | **Gene Bank** | **Primer sequence** | **cDNA size (bp)** |
| --- | --- | --- | --- |
| VEGF-A | NM009505 | Forward: 5´-gctctcttgggtgcactgga-3' | 561 |
|  |  | Reverse: 5’ –caccgccttggcttgtcaca-3’ |  |
| FGF-2 | NM174056 | Forward: 5´-aactacaacttcaagcagaagagaga-3’ | 293 |
|  |  | Reverse: 5’ - ttaagatcagctcttagcagacat-3’ |  |
| TSP-1 | M62470 | Forward: 5´-cctcatttgttgtgtgactgagtaa-3’ | 556 |
|  |  | Reverse: 5’-ttgttgttccttgtacataagaaac-3’ |  |
| MMP-2 | M84324 | Forward: 5´-agatcttcttcttcaaggaccggtt-3’ | 224 |
|  |  | Reverse: 5´-ggctggtcagtggcttggggta-3’ |  |
| MMP-9 | NM_013599 | Forward: 5´-tgcgaccacatcgaacttcg-3’ | 683 |
|  |  | Reverse: 5´-gagaagaagaaaaccctcttgg-3’ |  |
| TIMP-1 | NM_001044384 | Forward: 5´-ggcatcctcttgttgctatcactg-3' | 170 |
|  |  | Reverse: 5’-gtcatcttgatctcataacgctgg-3’ |  |
| TIMP-2 | NM_021989 | Forward: 5´-gagatcaagcagataaagatg-3’ | 320 |
|  |  | Reverse: 5’-gacccagtccatccagaggc-3’ |  |
| ACOX | J02752 | Forward: 5’-actatatttggccaattttgtg-3’ | 195 |
|  |  | Reverse: 5’-tgtggccagtggtttccaagcc-3’ |  |
| Thiolase | AY273811 | Forward: 5’-aaatgggtcttatgacattg-3’ | 425 |
|  |  | Reverse: 5’-cactcacctgactggagttt-3’ |  |
| MACD | NM_007382 | Forward: 5’-gacatttggaaagctgctagtg-3’ | 321 |
|  |  | Reverse: 5’-tcacgagctatgatcagcctctg-3’ |  |
| VLCAD | AF017178 | Forward: 5’-cgtcagaggtgtactttgatgg-3’ | 269 |
|  |  | Reverse: 5’-catggactcagtcacatactgc-3’ |  |
| β-actin | NM_00793 | Forward: 5´-tggaatcctgtggcatccatgaaac-3´ | 348 |
|  |  | Reverse: 5´-taaaacgcagctcagtaacagtccg-3´ |  |
